# The Effect of Powder and Emulsion Binders on the Tribological Properties of Particulate Filled Glass Fiber Reinforced Polymer Composites

**DOI:** 10.3390/polym15010245

**Published:** 2023-01-03

**Authors:** Wojciech Zurowski, Jaroslaw Zepchlo, Robert Cep, Lenka Cepova, Miroslaw Rucki, Zbigniew Krzysiak, Jacek Caban, Waldemar Samociuk

**Affiliations:** 1 Faculty of Mechanical Engineering, Kazimierz Pulaski University of Technology and Humanities in Radom, ul. Stasieckiego 54, 26-600 Radom, Poland; 2 RADWAG, ul. Toruńska 5, 26-600 Radom, Poland; 3 Faculty of Mechanical Engineering, VSB-Technical University of Ostrava, 17. listopadu 2172/15, 70800 Ostrava, Czech Republic; 4 Institute of Mechanical Science, Vilnius Gediminas Technical University, J. Basanaviciaus Str. 28, LT-03224 Vilnius, Lithuania; 5 Faculty of Production Engineering, University of Life Sciences in Lublin, Głęboka 28, 20-612 Lublin, Poland; 6 Faculty of Mechanical Engineering, Lublin University of Technology, Nadbystrzycka 36, 20-618 Lublin, Poland

**Keywords:** polymer composite, glass fiber reinforcements, epoxy resin, tribology, wear resistance

## Abstract

Investigations into polymer composites are mainly focused on properties dependent on glass fiber reinforcement and particulate fillers. In the present study, the effect of the binder was examined. The specimens were produced with two types of epoxy resin, with similar numbers of glass mat layers and similar proportions of quartz powder added. However, one group was fabricated with an emulsion binder in the glass mats and another group with a powder binder. Attention was concentrated on the tribological properties of the as-prepared composites, though their strength was examined as well. The hardness of the Sikafloor matrix was found to be much more sensitive to the applied binder than that of the MC-DUR matrix. No direct correlation between the microhardness and the specific wear rate was observed and increasing the particulate filler proportion did not cause a direct increase of the specific wear rate. In particular, the highest specific wear rate, around 350 J/g, was reached for both matrices with a 1% quartz addition when the emulsion binder was applied, while in the case of the powder binder it was with 6% quartz with the MC-DUR matrix, and there was no quartz addition with the Sikafloor matrix. The highest microhardness, *HV*_0.5_ = 25, in turn, was reached for the mats with the emulsion binder in the Sikafloor matrix with an addition of 10% quartz powder, while the highest friction coefficient was exhibited in the composite with the MC-DUR matrix, when 1% of the quartz powder and the emulsion binder were applied.

## 1. Introduction

The main advantages of fiber-reinforced polymer composites, widely used, for decades, in various engineering applications, compared to traditional materials, include their high strength to weight ratio and a good fracture resistance along the direction of the fiber reinforcement [1,2]. For example, housings made out of a polymer composite had their weight reduced by more than 60%, compared to that of steel [3]. Some authors also emphasize the anti-corrosive properties and high tensile strength, which make fiber-reinforced polymer an attractive engineering and construction material [4].

Glass fiber is a type of reinforcing material that serves to produce fiber-reinforced polymeric composites, intended to be as strong as steel and at least as stiff as aluminum, and to have a relative density of one-fourth that of steel [5,6]. Polymer composites reinforced with E-type glass fibers, due to their light weight, are widely used in the aerospace, marine, and other industries. The constituent elements in E-type glass fiber composites are silica (SiO_2_), alumina (Al_2_O_3_) and the oxides of calcium (CaO), magnesium (MgO), and boron (B_2_O_3_) [7]. E-glass owes its popularity as a fiberglass to its low density, better strength and greater stiffness, its significant heat resistance, fire resistance, and a better endurance to chemicals, as well as the ability to keep its structural integrity in unfavorable circumstances [8]. The comparison with similar polymer composites with basalt and glass fibers reveals a similar performance in many aspects, in particular, an increase in the amount of reinforcement in the composite material which always results in an improvement of the thermal conduction [9].

In agreement with the materials science methodology, the development of new composites with a reduced wear rate includes testing some new combinations of different fillers and reinforcements with a given polymer matrix [10]. For example, Xian et al. investigated a carbon-glass fiber reinforced polymer composite with random fiber hybrid and core-shell hybrid plates. The authors demonstrated that the random fiber hybrid plate exhibited an outstanding corrosive resistance and increased tensile strength by 51.3%, while the flexural strength increased by 39.7% [11]. Sharma and Gupta investigated the chemical resistance of an E-glass fiber reinforced epoxy resin composite with particulate fillers of aluminum oxide and silicon carbide, demonstrating a strong chemical resistance of 2 wt.% Al_2_O_3_/SiC (1:1) filled composites against acids and solvents for one day [12]. Raj et al. [13] examined hybrid polymer composites fabricated using natural and synthetic fibers, including glass, hemp, abaca, and Kevlar. They reported that the tensile properties of Kevlar/abaca and glass/hemp composites, compared to glass/abaca materials, were higher by 267.74% and 6.45%, respectively. Moreover, the impact energy absorbed by the Kevlar/abaca and glass/hemp composites was found to be higher, by 63.34% and 55.74%, respectively, than that of the glass/abaca composite, and the Kevlar/abaca composite exhibited the maximal strength in the tensile, flexural, and impact tests. Kobyliukh et al. [14] demonstrated that the introduction of hybrid nanoscale fillers, composed of graphene materials and some different structures of iron oxides to the polymer matrix, provided the new materials with unique characteristics due to their ability of self-organizing in the polymer matrix under an external magnetic field.

Newly developed polymer composites must undergo a reliability assessment to identify hazards and to undertake proper safety measures [15]. Figlus and Kozioł [16] proposed applying vibration signals for an early-stage damage diagnosis of polymer glass fiber reinforced composites. In fact, the defect detection is important, as early as at the machining stage [17]. The tribological characteristics of the polymer composites are also highly important [18].

Addressing the ecological issues, an interesting report on the fabrication method and properties of the natural rubber bio-composites reinforced with cereal straw powder modified with functional nanoscale additives was published [19]. The authors demonstrated that the cereal straw reinforced the elastomer matrix, contributing to the good mechanical properties of the composite obtainable at a low cost. Much attention is paid to the recyclability of fiber polymer composites, but most of the reviewed research concentrates on the fiber phase recovery, while the polymeric phase is commonly downcycled to fuel [20]. Somaiah et al. [21] performed a comprehensive review exploring the mechanical and thermal properties dependent on the chemical composition of glass fiber reinforced polymer composites and of their constituents. They also undertook a brief review of various polymer matrix materials and filler materials. The effect of the glass fiber reinforcement and particulate filler is examined by many researchers. For instance, Elfarhani et al. [22] examined the influence of the laminating configuration of a glass fiber-reinforced polymer, during the dry edge trimming process. Sattar et al. [23] tested composites made of a 1″ long glass fiber, incorporated within a polyamide 6 (nylon) matrix. Mao et al. demonstrated that the performance of 28% gear pairs was significantly enhanced after the addition of the glass fiber reinforcement, compared to non-reinforced pairs, but after the transition load was reached, the thermal wear took place with the local surface melting [24]. Muhammad et al. [25] added eggshell fillers to glass fiber reinforced polymer composites to improve their tensile and flexural characteristics. Another research team reported their results on nano-silica particles added as secondary reinforcement to a glass fiber reinforced epoxy resin matrix composite, thereby enhancing its strength [26]. In turn, Thiagarajan and others [27] investigated the impact of pistachio shell particles added to the mechanical properties of a glass fiber reinforced epoxy polymer composite.

However, while there are many papers concerning the glass fiber reinforcement and particulate filler [28,29], it seems that no research has been dedicated to the effect of a binder application during the epoxy resin preparation. Therefore, we have undertaken a novel research program aimed at a comparison of the commonly available materials, yet taking into account the usually omitted detail, namely, the form of a binder, i.e., a powder or emulsion. Considering the specific wear rate as the most important parameter for this specific purpose, we used a novel method for the tribological tests. Usually, ball-on-disc [30] or pin-on-disc [31] systems are utilized, but it is widely admitted that debris left in the friction area may distort the results [32,33]. The novel tribological tester TT-4, used in our experiment, was free of this disadvantage.

In the present study, the effect of the application of a powder binder and epoxy binder in the fabrication of similar glass fiber reinforced composites with the same proportions of a quartz particulate filler were examined. The attention was focused on the tribological properties of the composites, although the material strength was taken into consideration as well. The research was aimed, in particular, at determining the most proper composition for the further fabrication of a truck floor covering. In these conditions, the floor material mainly undergoes a sliding wear test under different mechanical loads over relatively short distances, when a truck is loaded or in motion. Relatively low sliding velocities are expected, below 180 mm/min, so that the stick-slip motion can be present [31]. Moreover, the experiments would provide the practical guidance on how to use the available components, to obtain the most desired performance of the composite. Thus, the investigations focused on the purpose-oriented tribological properties, which were focused on a relatively cheap composite material with an optimal achievable wear resistance.

## 2. Materials and Methods

### 2.1. Tested Samples

The tested materials were based on two types of commercially available resins, MC-DUR 1200VK, fabricated by MC-Bauchemie Müller GmbH (Bottrop, Germany) and Sikafloor 156, produced by Sika Deutschland GmbH (Stuttgart, Germany), respectively. According to the specifications [34,35], both materials exhibited a similar wear resistance ≤ AR1, but different impact strengths: IR4 for MC-DUR and IR10 for Sikafloor (EN 13813:2002). Both materials had the same density of 1.1 g/cm^3^. Unfortunately, the data on flexural and compressive strength given in the specifications was for 7 days in the case of MC-DUR resin, while in the case of Sikafloor it was for 30 days.

Both composite matrices were reinforced with four layers of E-type EM1004 glass mats supplied by Krosglass S.A. (Krosno, Poland), with a mass per unit area 300 g/m^2^ that exhibited a modulus of 76 GPa and the tensile stress of 3.45 GPa, according to the literature [36]. The mats were made of 12 µm diameter glass fibers with a linear weight of 30 tex and the length of ca. 50 mm. The arrangement of the fibers was random. The glass mats were available in two variants: one fabricated using an emulsion binder and another fabricated with a binder in the form of a powder. As suggested by the experimental research, the form of the binder used in the mats had some effect on the final tribological characteristics of the composites.

The particulate filler was added, in the form of a quartz powder consisting of 99.57% SiO_2_, 0.12% Al_2_O_3_, and 0.31% of other oxides. The dimensions of the quartz particles were in the range between 0.1 mm and 0.3 mm. Based on previous experience, the proportions of the particulate filler in the samples, by mass, were 0%, 1%, 3%, 6%, and 10%. To allow for a statistical analysis of the tribological characteristics, six samples of each composition were made. Table 1 presents the basic information on the tested polymer composite samples.

**Figure 1:**
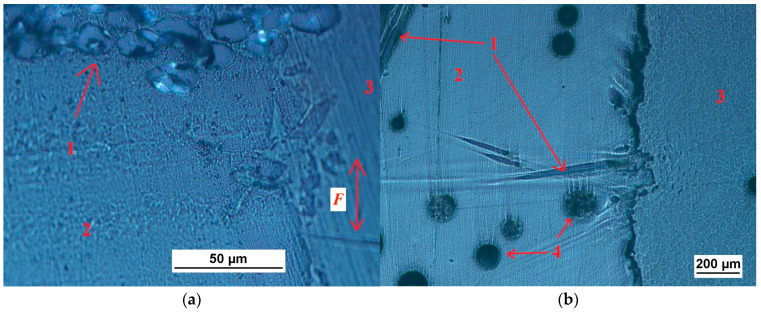
The examples of the specimens: (**a**) made of MC-DUR 1200VK epoxy resin with an emulsion binder glass reinforcement and no addition of quartz powder; (**b**) made of Sikafloor 156 epoxy resin with a powder binder glass reinforcement and the addition of 6 wt.% quartz powder. Explanations: 1—glass fibers, 2—resin, 3—epoxy for inclusion, 4—air bubbles (pores), *F*—direction of friction forces.

To prepare the laminates, the hand lay-up method was applied. The epoxy resin was mixed with a hardener in the proportion of 3:1, by weight. A vibration table was used to reduce the porosity. Following the lamination of four layers, the plates were pressed under a pressure of 2 MPa and then hardened for 24 h at room temperature. However, the as-prepared specimens underwent experimental tests no earlier than 2 weeks after their fabrication. Figure 1 presents some examples of the microstructures of the different specimens.

### 2.2. Tribological Tests

The wear resistance and tribological performance of a solid surface, is a feature that depends on the properties of the counter-faces involved in the system, including the hardness and roughness [37]. It is well established that the tribological characteristics of plastics and elastomers are dependent on the test pressure and velocity, as well as on the plastic material finish, the surface geometry and finish, the ambient temperature, the mating surface hardness, and the thermal conductivity [38]. An increase of the surface hardness improves the wear resistance in the abrasive contact with hard particles [39]. Thus, checking the hardness of the specimens presented in Table 1 was found to be important. The microhardness *HV*_05_ was measured using a Wilson 401 MVD Knoop/Vickers microindentation tester produced by Buehler (Lake Bluff, IL, USA).

The specimens for the tribological tests were of 30 mm height × 15 mm width × 2.8 mm thickness. This form was designed specifically for the dedicated tribotester TT-4, used in the research, and described in detail elsewhere [40]. It was proven to ensure the repeatability of %*EV* = 13.4%, while the typical pin-on-disc tribological tester T-01M exhibited a much higher dispersion of the results with %*EV* = 33.7%. The most important feature of the TT-4 device was a moving friction belt capable of removing the worn material of a sliding specimen, so that the worn surfaces and the debris were not involved in the test and thus did not bias the results. As a result, the mating surface is maximally unified and not affected by the test itself, while the 50 m long sliding path ensured the stability of the results. Moreover, the moving friction belt stabilized the temperature in the contact area, continuously removing the heated debris and providing a new abrasive surface. Using the registered data, the average friction force was calculated as *F_fsr_* (N) for each sample, as well as for the friction work *A_f_*.

The tribological tests were performed for 60 s for each sample, at a friction belt velocity of 0.2 ± 0.01 m/s, and under a pressing force of 250 ± 12.5 kPa. Given those conditions, the sliding distance was *s_d_* = 12 m. The ambient temperature of 23 ± 1 °C and the air humidity between 40% and 50%, were maintained. The friction resistance was recorded with an accuracy of ±0.1 g, and the sampling frequency was 2 Hz.

Since it was necessary to detect the outliers in the small number of experiments, it was found to be appropriate to apply Dixon’s *Q* test, as detailed in the literature [41]. According to the procedure, the respective minimal *Q*_min_ and maximal *Q*_max_ observations were calculated as follows, based on the results ranked from the lowest *x*_1_ to the highest *x*_n_:

*Q_max_* = (*x_n_* − *x_n−_*_1_)/(*x_n_* − *x*_1_), (1)

*Q_min_* = (*x_2_* − *x*_1_)/(*x_n_* − *x*_1_). (2)

The largest *Q* values were then compared with *Q_kr_*, considered to be a critical value for the given number of repetitions and the assumed level of confidence [41]. In our case, for a 95% confidence and 10 measurements, *Q_kr_*_10_ was 0.412, while for six measurements, it was *Q_kr_*_6_ = 0.560.

### 2.3. Additional Tests

**Figure 2:**
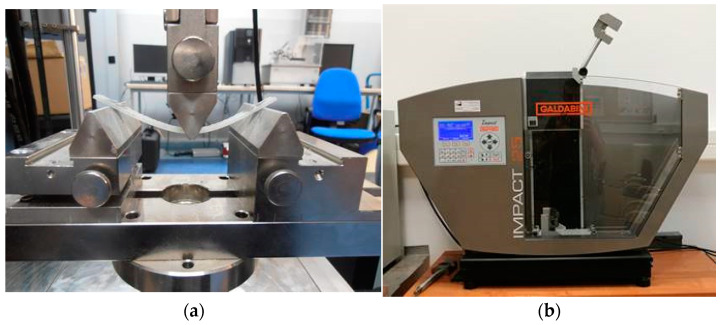
The examples of equipment used in the experiments: (**a**) flexural strength measurement with Zwick/Roell Z100; (**b**) impact strength measurement with Impact 25.

Since the presumed application of the tested composites was in the floor panels, it was decided to focus on the wear resistance rather than on the tensile strength. The flexural strength, microhardness, shear strength, and impact strength were measured, according to the following standards: DIN EN ISO 14125, EN ISO 6507-1:1997, PN-EN 2377:1994, and PN-EN ISO 179-1:2010, respectively. Figure 2 shows some examples of the flexural strength measurement with a Zwick/Roell Z100 device (ZwickRoell GmbH & Co., Ulm, Germany) and the impact strength measurement with the Impact 25 device, made by Galdabini (Cardano al Campo, Italy).

The Zwick/Roell Z100 device also made it possible to perform interlaminar shear strength tests. The changing distance between the supports was the difference compared to the bending test. The test was conducted with the speed of 20 mm/min, the radiuses of the supporting pins were 2 mm, and that of the bending pin was 5 mm. The resulting *τ*_max_ (MPa) indicated the resistance of the laminate to the interlaminar shear load.

## 3. Results and Discussion

This section presents the results generated for the microhardness, friction coefficient, specific wear rate, and strength of the tested composites. To identify the effect of a binder, the results are shown in diagrams grouped for the composites with the emulsion binder and the powder binder, respectively.

### 3.1. Microhardness

**Figure 3:**
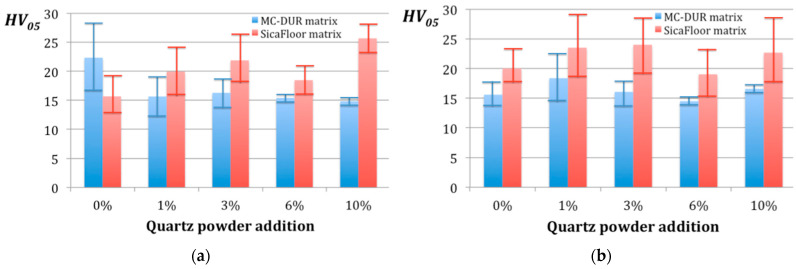
Microhardness *HV*_05_ dependent on the particulate filler proportions for the composites with the polymer matrices MC-DUR 1200VK and Sikafloor 156: (**a**) with the emulsion binder; (**b**) with the powder binder.

The effect of the binder on the composite hardness was different for both tested matrices. Figure 3 presents the diagrams of the microhardness *HV*_05_ dependent on the particulate filler proportions for each respective composite.

First of all, it should be noted that *HV*_05_ of the composites, based on the MC-DUR matrices, remained almost unaffected, by both the binder type and by the particulate filler proportions. Its average value remained between 14 and 18, with only one exception of the emulsion binder with no quartz powder added, which reached *HV*_05_ = 22.33 (Figure 3a).

It appeared that the Sikafloor matrix was much more sensitive to the binder applied. When comparing the respective microhardnesses of the composites with the different contents of the quartz powder, it can be seen that 0–6% of the particulate filler increased the hardness of the composites with the powder binder. In turn, a higher proportion of 10% quartz powder in the composite with the emulsion binder resulted in the highest hardness among all of the tested materials. Notably, a high hardness above *HV*_05_ = 23 was reached by the Sikafloor matrix composites with the powder binder and with the quartz powder additions of 1% and 3%.

The addition of 6% quartz powder seems disadvantageous for of all the tested composites, from the perspective of the microhardness. This finding is very important and indicates that the proportion of a 6% particulate filler is not recommended for the four-layer glass fiber reinforced composites of this type, due to the loss of hardness.

### 3.2. Friction Coefficient

It was difficult to evaluate the impact of the particulate filler percentage and the binder applied on the friction coefficient of the tested samples, due to a large dispersion of the results. Despite the fact that the tribological tester TT-4 provided for a minimization of the undesired effects on the measurement results, and exhibited a much better repeatability than the pin-on-disc devices, the standard deviation of the results reached several percent of the measured friction coefficient. Table 2 contains the results obtained for six specimens of Sikafloor matrix composites with the powder binder. The large standard deviation values can be attributed to the complexity of composite structures.

**Figure 4:**
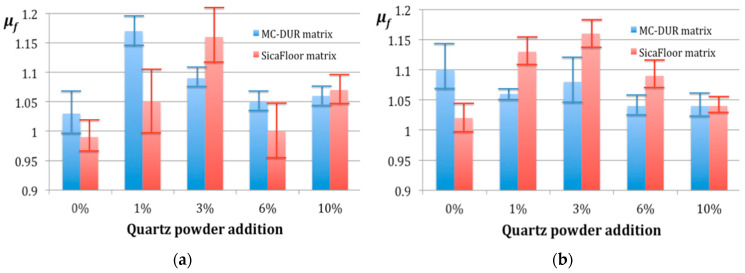
The average values of the friction coefficient *μ_f_* dependent on the particulate filler proportions for the composites with the polymer matrices MC-DUR 1200VK and Sikafloor 156: (**a**) with the emulsion binder; (**b**) with the powder binder.

It should be noted that the difference between the largest and smallest average friction coefficients *μ_f_* in this tested group was 0.16, roughly three times the standard deviation registered for the specimens with no particulate filler (0% of quartz powder added). Thus, most of the differences between the coefficients *μ_f_* assigned to the particular composites may be explained by the statistical error. Nevertheless, some conclusions may be derived from the average values of the results, as presented in the diagrams in Figure 4.

First of all, it can be stated that the Sikafloor matrices are more sensitive to the percentage of the particulate filler. The composites made using the emulsion binder, with both polymer matrices MC-DUR 1200VK and Sikafloor 156, exhibited larger differences between the highest and the lowest friction coefficients *μ_f_*, 0.14 for the MC-DUR and 0.17 for the Sikafloor matrices.

Another interesting observation is that the Sikafloor matrices were less sensitive to the binder type. Both of them, treated with either the emulsion or the powder binder, exhibited the highest friction coefficient *μ_f_*_max_ when the proportion of the particulate filler was 3 wt.% and the lowest was without any filler. Unlike the Sikafloor matrices, MC-DUR was more sensitive to the binder type. In particular, when the emulsion binder was applied, the minimal friction coefficient corresponded to no quartz powder addition, while the maximal one *μ_f_*_max_ was recorded when the proportion of the particulate filler was 1 wt.%, and was further reduced with an increasing proportion of quartz powder. Moreover, when the powder binder was applied to the MC-DUR matrices, the difference between the average *μ_f_* values *μ_f_*_max_ = 1.10 and *μ_f_*_min_ = 1.04 was 0.06, close to the standard deviation value. Thus, it cannot be considered statistically significant. However, we refer to the result reported by Walczak et al. [42], where the composite exhibited a friction coefficient lower by 0.04, than that of the matrix material. It stands in general agreement with the observation made by Rajak et al. that the addition of fillers, including glass fibers, tends to enhance the tribological behavior of the polymeric matrix composite by diminishing the coefficient of friction [43].

To sum up, some three important conclusions can be derived concerning the friction coefficient. Firstly, the MC-DUR matrices, treated with the powder binder, exhibited the smallest sensitivity to the particulate filler content. Secondly, the Sikafloor matrices tended to exhibit the highest friction coefficient when the quartz powder filler was added in the proportion of 3 wt.%, for both the emulsion and powder binders. Finally, a similar value of the friction coefficient could be reached with the MC-DUR matrix, only when the emulsion binder was applied and 1 wt.% of the quartz powder was added.

### 3.3. Specific Wear Rate

**Figure 5:**
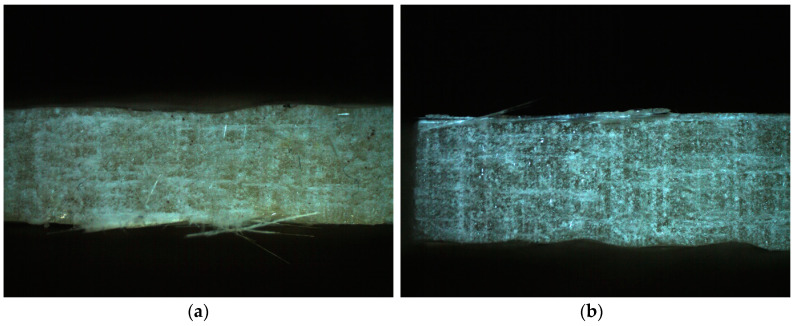
Some examples of the worn composite surfaces with the polymer matrices MC-DUR 1200VK and the particulate filler proportions of 10 wt.%: (**a**) with the emulsion binder; (**b**) with the powder binder.

**Figure 6:**
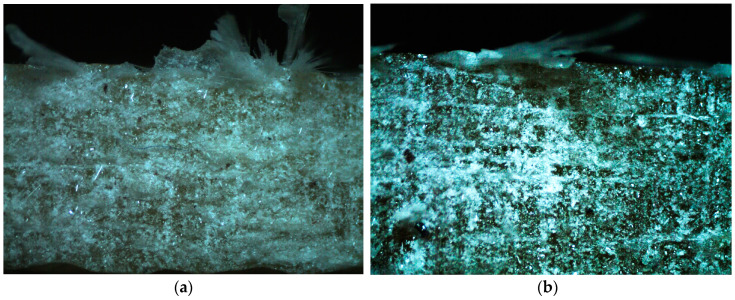
Some examples of the worn composite surfaces with the polymer matrices Sikafloor 156 with the particulate filler proportions of 10 wt.%: (**a**) with the emulsion binder; (**b**) with the powder binder.

Following the tribological tests, the worn surfaces were thoroughly analyzed. There are many reports on the effects of the test parameters, such as the erodent type, the size of the eroding particles, the impact velocity, and the impingement angle on the wear rate of the polymer composites [44]. To standardize the results, the specific wear rate was calculated. In our case, the main wear mechanism turned out to be abrasive, which can be defined in general terms as ‘the loss of material by the passage of hard particles over a surface’ [45]. Abrasive wear is usually caused ‘by particles that are embedded in or attached to some opposing surface’ [46], which was the case in the present study, as is clearly seen in Figure 5 and Figure 6. Moreover, some delaminations can be observed, especially at the larger multiplications, as in Figure 6. The lack of deformation areas indicates a negligible effect of the friction forces on the composite’s inner layers. In other words, the work of the mechanical interaction between the two contacting surfaces was consumed mainly by the removal of particles from the specimen. A few grains were found torn out of the friction belt after the test.

**Figure 7:**
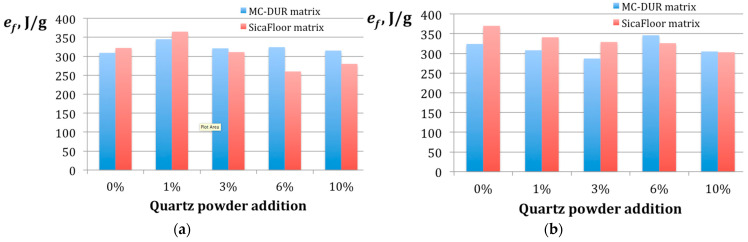
The average values of the specific wear rate *e**_f_*** obtained for the specimens of the different particulate filler proportions in the polymer matrices MC-DUR 1200VK and Sikafloor 156, produced using different binders: (**a**) with the emulsion binder; (**b**) with the powder binder.

Considering the wear mass Δ*m*(g) lost by the specimen during the test, the specific wear rate *e**_f_*** (J/g) was calculated as follows:

*e_f_* = (*F_fsr_* × *s_d_*)/Δ*m*, (3)

where *F_fsr_* is the average friction force (N) and *s_d_* is a sliding distance (m) during the test. The diagrams of the specific wear rate values for the tested composites are presented in Figure 7. For the sake of comparison, the specific wear rate for the other materials was measured and it was *e_f_* = 3050 J/g for steel, *e_f_* = 1681 J/g for aluminum, and *e_f_* = 1166 J/g for wood.

The impact of a binder used in the fabrication of the tested polymer matrix composites on the specific wear rate *e**_f_*** did not exhibit any clear trend. Presumably, the irregular effect of the increased quartz powder can be explained by the uneven load distribution between the filler, glass fiber reinforcement, and matrix during the wear tests [47]. However, some important observations can be derived from the diagrams shown in Figure 7.

In the case of the emulsion binder, a distinct maximal wear resistance is indicated by *e**_f_*****_max_**≈ 350 J/g, reached by the composites with both the MC-DUR and Sikafloor matrices, including 1 wt.% of the particulate filler. The difference between the matrices can be seen in the fact that the MC-DUR-based composites maintained their *e**_f_*** above 309 J/g, while the Sikafloor-based composites lost their wear resistance below *e**_f_*** = 300 J/g when the 6 wt.% and a higher proportion of the quartz powder was added.

When the powder binder was used for the fabrication of the composites, the Sikafloor matrices exhibited an almost linear decrease of their wear resistance from *e**_f_*** = 369 J/g, corresponding to 0% of the particulate filler, down to *e**_f_*** = 302 J/g for 10 wt.% of the quartz powder. In turn, the MC-DUR-based matrices exhibited specific wear rate *e**_f_*** values between 187 and 324 J/g with only one exception. Namely, the 6 wt.% addition of the quartz powder caused an increase of *e**_f_*** to 346 J/g.

Notably, no direct correlation between the microhardness *HV*_05_ and the specific wear rate *e**_f_*** can be distinguished. The only composition that kept the maximal values of both the hardness and specific wear rate in its group, was the MC-DUR matrix with 1 wt.% of the particulate filler, prepared using the emulsion binder.

To sum up, since the abrasive wear was the dominating mechanism in the tested specimens, the specific wear rate can be considered the main indicator of the wear resistance. In particular, an increase in the particulate filler in the form of quartz powder did not cause a direct increase of the specific wear rate. A clear correlation between a high microhardness and a high wear resistance was found in one composition only. The maximal wear resistance was reached by the composites with both the MC-DUR and Sikafloor matrices and 1 wt.% of the particulate filler. The Sikafloor-based composites exhibited a higher sensitivity to the percentage of the quartz filler added.

### 3.4. Strength

**Figure 8:**
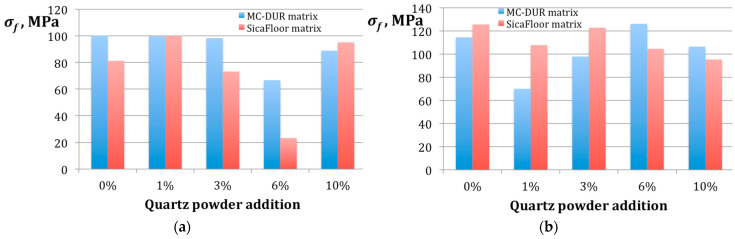
The results of the flexural strength *σ_f_* measurement for the specimens of the different particulate filler proportions in the polymer matrices MC-DUR 1200VK and Sikafloor 156, produced using different binders: (**a**) with the emulsion binder; (**b**) with the powder binder.

The results for the flexural strength, shear strength, and impact strength, did not exhibit a direct correlation with the particulate filler proportion, either, as it would have been expected. Figure 8 presents the results of the flexural strength measurement.

The flexural strength of the specimens with the Sikafloor matrix produced using the emulsion binder with the addition of the 6 wt.% quartz powder proved exceptionally small. However, the test involved 10 specimens and the standard deviation between the results was 1.08, below 10% of the measured value. Thus, if there was an excessive error, it covered the entire group of the specimens. It should be noted that some decrease in the flexural strength took place in the case of the MC-DUR-based composites with a similar proportion of quartz powder, so it can be concluded that the emulsion binder might have been sensitive to the 6 wt.% particulate filler. This assumption could be confirmed by the fact that the MC-DUR composite made with the powder binder exhibited the highest flexural strength at the same 6 wt.% proportion of the quartz powder addition. Moreover, the maximal *σ_f_* obtained for both the matrices, corresponded to the powder binder and was ca. 25% higher than that obtained with the emulsion binder.

There are reports that comparisons in the flexural strength with the wear resistance of the polymer materials scraped to the same thickness revealed a correlation between the changes in the flexural strength and the material surface deterioration [48]. In our research, this correlation is not so obvious. A comparison of Figure 7a and Figure 8a indicates that, to some extent, when the emulsion binder was used, 1 wt.% of the quartz powder resulted with the highest values of both the flexural strength and the specific wear work. For the powder binder, clear peaks do not correspond to one another, though it may be stated that 0% and 6% of the quartz filler addition produced high values in the flexural strength and specific wear rate. It may be explained by the fact that the flexural load caused the stretching tension in the outward layers of the specimen and the compression in its inward part. Tilak et al. [49] suggested that in the specimens of different compositions, a shear load could dominate, resulting with the failure of the matrix.

**Figure 9:**
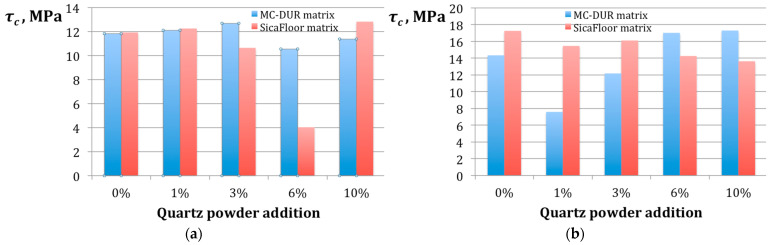
The results of the shear strength *τ_c_* measurement for the specimens of the different particulate filler proportions in the polymer matrices MC-DUR 1200VK and Sikafloor 156, produced using different binders: (**a**) with the emulsion binder; (**b**) with the powder binder.

The shear strength of the fiber reinforced polymer composites is strongly dependent on the polymer matrix and it is generally agreed that the shear strength of a composite is limited by the shear strength of the matrix [50]. The interlaminar shear strength is especially vital for many applications of polymer composites [51]. Our research demonstrated some effect of the particulate filler addition and the applied binder. Figure 9 presents the results of the shear strength *τ_c_* measurement for the tested composite specimens.

Generally, the pattern of the shear strength seen in Figure 9, is similar to the one of the flexural strength in Figure 8. This may be explained by the dominating abrasive wear mechanism with a rather small degree of delamination. It should be noted that the composites made with the emulsion binder were unable to reach a shear strength *τ_c_* = 13 MPa, while most of the results for the powder binder were above 14 MPa. In particular, the composites with the Sikafloor-based matrices all exhibited *τ_c_* > 13 MPa.

The crucial role of the binder can be seen from the comparison of the patterns of blue bars in Figure 9a,b. They indicate that the emulsion binder could provide the highest shear strength with 0% to 3% of the particulate filler addition, unlike the powder binder, which reached the highest values of *τ_c_* for the compositions with 6% and 10% of the quartz powder.

**Figure 10:**
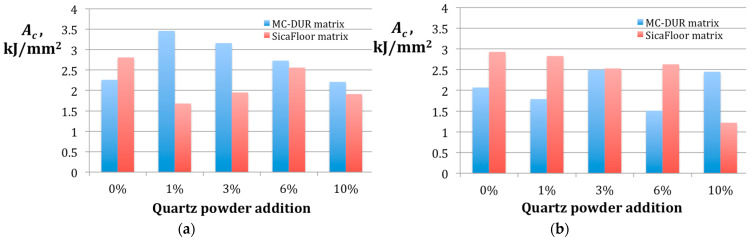
The results of the impact strength *A_c_* measurement for the specimens of the different particulate filler proportions in the polymer matrices MC-DUR 1200VK and Sikafloor 156 produced using different binders: (**a**) with the emulsion binder; (**b**) with the powder binder.

According to [52], the particles of filler homogeneously mixed with the resin improve the interfacial bond between the matrix and the fibers. In addition, the particulate filler in the resin resists the micro-crack propagation, providing an improvement of the impact strength. It can be expected that a higher amount of the filler added to the resin would result in a higher impact strength [52]. In our study, that was not the case. The impact strength results, representing the absorbed energy per cross section upon the catastrophic fracture [53], are shown in Figure 10.

It is seen from Figure 10a that the highest impact strength of *A_c_* = 3.46 kJ/mm^2^ was reached for the MC-DUR matrix-based composite treated with the emulsion binder, containing 1 wt.% of the quartz powder filler. Increasing the quartz powder proportion caused a weakness of the composites and when the particulate filler content was 10 wt.%, the impact strength was *A_c_* = 2.21 kJ/mm^2^, even lower than that for the composite without any quartz powder added. In the case of the Sikafloor composites treated with the emulsion binder, Figure 10a shows that its maximal impact strength *A_c_* = 2.81 kJ/mm^2^ was produced with no addition of the particulate filler.

Interestingly, the composites based on the Sikafloor matrices treated with the powder binder exhibited an almost reverse effect to that suggested by [53]. The red bars in Figure 10b correspond to the impact strength reducing as the percentage of the quartz powder rises. The only exception was *A_c_* = 2.63 kJ/mm^2^ for 6 wt.% of the particulate filler, but it differed from the value *A_c_* = 2.53 kJ/mm^2^ for 3 wt.% only by 0.1 kJ/mm^2^, while the standard deviation for the set of 10 specimens was ca. 0.4 kJ/mm^2^.

## 4. Conclusions

The main conclusion suggested by the results is that the form of a binder, either emulsion or powder, has some effect on the performance of the epoxy resin-based composites with a glass fiber reinforcement and a particulate filler. Considering the friction coefficient, the MC-DUR matrices treated with the powder binder exhibited the smallest sensitivity to the particulate filler content. In turn, the Sikafloor matrices tended to exhibit the highest friction coefficient when the quartz powder filler was added in proportion of 3 wt.%, for both the emulsion and powder binders. Moreover, a similar value of the friction coefficient could be reached with the MC-DUR matrix, only when the emulsion binder was applied and 1 wt.% of the quartz powder was added.

From the perspective of the microhardness, it appeared that the Sikafloor matrix was much more sensitive to the binder applied. The addition of 0–6% of the particulate filler increased the hardness of the composites fabricated with the powder binder. In contrast, it was 10% of the quartz powder in the composite with the emulsion binder, which produced the highest hardness obtained in all of the tested materials.

The impact of a binder applied to the fabrication of the tested polymer matrix composites on specific wear rate did not exhibit any clear trend. It should be noted that, in the case of the emulsion binder, a distinct maximal wear resistance *e**_f_*****_max_** ≈ 350 J/g was reached using the composites with both the MC-DUR and Sikafloor matrices containing 1 wt.% of the particulate filler. When the powder binder was used, the Sikafloor matrices exhibited an almost linear decrease of the wear resistance from *e**_f_*** = 369 J/g, corresponding to 0% of the particulate filler, down to *e**_f_*** = 302 J/g for 10 wt.% of the quartz powder. In turn, the MC-DUR-based matrices exhibited specific wear rate *e**_f_*** values between 187 and 324 J/g, with only one exception. Namely, a 6 wt.% addition of the quartz powder increased *e**_f_*** to 346 J/g.

As for the impact strength, the composites, based on the Sikafloor matrices treated with the powder binder exhibited an almost opposite effect to what would be expected. Namely, increasing the percentage of the quartz powder filler reduced the trend of the impact strength.

Considering an almost exclusively abrasive wear mechanism with a very small impact of delamination, future research will be focused on the adhesion between the glass fiber reinforcement and the matrix. Presumably, it will be possible to attribute some effect of the binder type in the polymer composite to the wettability and adhesive characteristics.

## Figures and Tables

**Table 1:** The compositions of the tested epoxy resin composite samples.

Matrix	Binder	Quartz Powder Filler Percentage, %				
MC-DUR 1200VK	Emulsion	0	1	3	6	10
	Powder	0	1	3	6	10
Sikafloor 156	Emulsion	0	1	3	6	10
	Powder	0	1	3	6	10

**Table 2:** The values of the friction coefficient *μ_f_* obtained for the Sikafloor matrix composites made using a powder binder, with the different percentages of the particulate filler.

Sample No.	Quartz Filler Proportion				
	0%	1%	3%	6%	10%
1	1.0787	1.0989	0.9207	1.0412	1.0584
2	0.9306	1.1974	1.0625	1.1310	1.0157
3	0.9996	1.0579	0.9107	1.0894	1.0361
4	1.0220	1.0970	0.9220	1.0334	1.0675
5	1.0093	1.1517	0.9929	1.1479	1.0533
6	1.0546	1.1611	1.0165	1.0931	1.0286
Average	1.02	1.13	0.97	1.09	1.04
Standard deviation	0.05	0.05	0.06	0.05	0.02
Percent standard deviation	5.03%	4.56%	6.43%	4.24%	1.89%

## Data Availability

Data available upon request.
